# Relationship between Beef Quality and Bull Breed

**DOI:** 10.3390/ani13162603

**Published:** 2023-08-12

**Authors:** Piotr Kostusiak, Jan Slósarz, Marcin Gołębiewski, Tomasz Sakowski, Kamila Puppel

**Affiliations:** 1 Institute of Animal Science, Warsaw University of Life Sciences, Ciszewskiego 8, 02-786 Warsaw, Poland; piotr_kostusiak@sggw.edu.pl (P.K.); jan_slosarz@sggw.edu.pl (J.S.); marcin_golebiewski@sggw.edu.pl (M.G.); 2 Institute of Genetics and Animal Biotechnology, Polish Academy of Science, Jastrzębiec, Postępu 36A, 05-552 Magdalenka, Poland; t.sakowski@igbzpan.pl

**Keywords:** cattle, beef, bioactive components, fatty acid profiles, antioxidants

## Abstract

**Simple Summary:**

The aim of this study was to determine and compare the nutritional value of meat from the four most popular cattle breeds in Poland. A study comparing the nutritional value and quality of beef from the Polish Holstein-Friesian (PHF) dairy breed with that from the Limousine (LM), Hereford (HH) and Charolaise (CH) beef breeds found that beef from beef breeds had higher levels of total antioxidant status (TAS) and degree of antioxidant potential (DAP) than PHF beef. The LM breed had the highest concentration of DAP, anserine, taurine, and creatine, while CH had the highest levels of TAS, carnosine, and coenzyme Q10. In addition, LM, CH, and HH had significantly higher levels of C18:2 *cis*-9, *trans*-11. The breed significantly influences the antioxidant potential of beef.

**Abstract:**

The beef industry in Poland heavily relies on the Polish Holstein-Friesian (PHF) breed, known for its primary use in dairy production, but which also contributes significantly to the beef supply. In contrast, the Limousine (LM), Hereford (HH), and Charolaise (CH) breeds have gained popularity due to their ideal specialized characteristics for beef production. As PHF continues to dominate the beef market, a thorough comparison of its beef quality and nutritional attributes with the three most popular beef breeds in Poland is essential. This study aims to address this knowledge gap by conducting a rigorous comparison. The experiment was carried out on the beef from 67 bulls kept in a free-stall system with standardized feeding. The highest total antioxidant status (TAS) was found in CH and was 147.5% higher than that in PHF. Also, compared with PHF, a large difference of 70% was observed in LM, while in HH it was only 6.25%. For degree of antioxidant potential (DAP), the highest concentration was found in LM, while CH had a slightly lower score than LM. PHF had the lowest scores for each of the analyzed parameters of protein fraction. For anserine, taurine, creatinine, and creatine content, the highest results were found for LM. For carnosine and coenzyme Q10, the highest values were found for CH. Overall, these results highlight the impact of maturity and breed on carcass composition and quality. Late-maturing breeds, such as LM and CH, tend to exhibit leaner carcasses with superior fatty acid profiles and antioxidant properties. This knowledge is valuable for producers, enabling them to make informed decisions regarding breed selection and production strategies to meet specific market demands for beef with the desired composition and quality.

## 1. Introduction

Cattle production in Poland is focused on milk production [1]. This means that breeding work carried out over the years has focused on improving animals for milk, especially in the Polish Holstein-Friesian (PHF) breed, and has ignored issues to do with the meat quality of these animals; this has been influenced by the lack of tradition related to beef consumption [2]. According to Statistics Poland (GUS), in 2021 there were 6,378,742 head of cattle, including 2,289,025 cows [3]. The number of beef cows under performance evaluation was determined by the Polish Federation of Cattle Breeders and Dairy Farmers (PZHiPBM), and on 31 December 2021 this figure was 21,840 head of cattle [4]. It was established that the average number of cows per farm was 21.8 head, and the number of herds registered with the PZHiPBM was 1147. The Polish Federation of Cattle Breeders and Dairy Farmers reported that there were 704,506 head of PHFs under dairy performance evaluation in 2021, which accounted for 88.76% of all dairy cows under evaluation in Poland and 11.04% of the total number of cattle [5]. The number of herds containing cows under performance evaluation in 2021 was 18,559. Thus, the proportion of beef cattle in Poland is dramatically low compared with the dominant dairy cattle population. This is due to milk production intensification, which results in dairy breeds dominating in beef production. In 2021, 1,866,484 bovine animals were slaughtered in Poland, including 52,053 calves, 310,881 heifers, 555,220 cows, and 948,330 bullocks and bulls [3], resulting in 553,706 tons in carcasses post-slaughter warm weight. Of the total amount of cattle in the country, 93% are dairy, so special attention should be paid to the quality and nutrient content indicators of the meat being produced, and crossbreeding should be considered as a way to improve these indicators [6]. Based on data since 2012, changes in the population of the most popular breeds have been observed. The changes in the Polish Holstein-Friesian (PHF), Limousine (LM), Charolaise (CH), and Hereford (HH) populations are shown in Table 1.

The characteristics of cattle production in Poland during the 2012–2021 period did not change significantly. Dairy production continued to be the dominant production sector. During this period, an increase of 17.86% in the country’s PHF herd, 17.4% in the LM herd, and 89.6% in the HH herd was observed, while the CH herd decreased by 30.6%, which is related to the higher price prosperity obtained for LM. The increases in the LM and HH herds are too low to be able to compensate for PHF beef production, which is the main contributor, so it is important to analyze and compare these breeds to determine their quality and health-promoting differences.

In order to study the influence of genotype on the formation of meat quality and its nutritional value, this study included bulls from the dominant dairy breed in Poland, the Polish Holstein-Friesian, and the three most popular beef cattle breeds, Limousine, Charolaise, and Hereford. The selection of breeds was made on the basis of available data on the proportion of breeds within the structure of beef production in Poland. The PHF breed is characterized by high milk yield, but in meat production it is characterized by poorer feed utilization and higher collagen content in muscles, which negatively affects consumption quality. Dairy cattle account for 93% of cattle heads, while the remaining 7% are made up of other cattle, including beef breeds, of which there are 15 registered in Poland. Of the beef breeds, about 70% are LM, 12% are CH, and 6% are HH. The cattle breeds used in the study make it possible to determine the quality of the meat produced in the country and to observe any differences due to the course of breeding work in Poland.

Beef is of worldwide importance as a source of protein fraction bioactive components such as anserine, carnosine, taurine, coenzyme Q10, creatine and creatinine, polyunsaturated fatty acids (FAs), fat-soluble vitamins, and of high-biological-value protein. All these elements have beneficial effects on human health [6]. Anserine and carnosine demonstrate antioxidant activity and inhibit the formation of carbonyl groups of proteins, which, by their actions, can prevent many diseases, including Alzheimer’s disease, atherosclerosis, and diabetes [7]. They also exhibit chelating and anti-glycation effects [8]. In the case the ω-3 family of fatty acids, C18:3 n-3, C20:5 n-3, C22:6 n-3, and C18:2 n-6, as well as C18:2 *cis*-9 *trans*-11, have anti-carcinogenic properties and antioxidant properties that help build a balance between oxidants and antioxidants. Fat-soluble vitamins also reduce the effects of free radicals [9]. β-Carotene and α-retinol affect the differentiation of epithelial cells in the gastrointestinal tract, urinary tract, respiratory tract, and organs for vision, and are essential for the biosynthesis of fat from sugars, as well as for catalyzing the oxidation of unsaturated fatty acids. α-Tocopherol has strong antioxidant properties and protects the body from degenerative diseases [9].

The production of high-quality culinary beef is still rare on Polish farms. Crossbreeding with beef breeds could be used to improve quality parameters and nutritional value, which would improve meat quality over a relatively short period of time, as well as feed conversion and growth rates [10]. Testing the concentrations of valuable nutrients will allow the necessary directions for animal breeding to be determined. Improving the analyzed parameters in the case of the PHF breed will benefit consumer health, improve meat quality through increased proportions of antioxidant substances [11], reduce the risk of animal diseases, and improve the export value of the raw material [12]. The aim of this study was to compare the beef quality and nutritional value of the dominant dairy breed and the three most popular beef breeds in Poland. The Polish Holstein-Friesian (PHF) breed is the main source of beef produced in the country. Limousine (LM), Hereford (HH), and Charolaise (CH) are the most popular beef breeds in Poland. A comparison of the four breeds in terms of beef quality will answer whether this is a good solution, especially when it comes to the formation of the level of bioactive components in muscle tissues. The PHF breed, which is the main source of the beef produced in Poland, was taken as the reference. The aim of this study was to determine and compare the nutritional value of meat from the four most popular cattle breeds in Poland

## 2. Materials and Methods

The experiment was conducted on 67 bulls from four breeds: Limousine, Hereford, Charolaise, and Polish Holstein-Friesian (PHF). Live weight and daily gain parameters were standardized at 605 days of age (Table 2). 

The characteristics of the feed characteristics are shown in Table 3. The bulls were kept in a free-stall system in accordance with the minimum standards for the maintenance of cattle (Journal of Laws No. 167/position 1629 of 2003, as amended).

The animals were slaughtered at 21–24 months of age, and the carcass weight was recorded before slaughter (selected groups in the same day). The carcasses were then cooled for 24 h at 2–4 °C, after which 300 g of semimembranosus muscles was sampled parallel to the muscle axis. During the fattening period, all the bulls received the same TMR ration, *ad libitum*, balanced according to National Research Council recommendations for beef cattle.

### 2.1. Analytical Methods 

Beef samples were chopped, then placed in a blender and ground until homogeneous. These was later analyzed using a near-infrared spectrophotometer. The basic chemical composition of the meat was determined using a Food Scan™ analyzer. 

Meat fat extraction was performed using the Folch method [13]. Fatty acid methylation was performed according to the EN ISO 5509 [14] transesterification method. The functional fatty acid content was determined using an Agilent 7890A GC gas chromatograph and a Varian Select FAME column according to Solarczyk et al. [6]. 

The measurement of the fat-soluble vitamin content was performed using an Agilent 1100 RP-HPLC instrument and a ZORBAX Eclipse XDB column according to the methodology of Puppel et al. [15]. 

The measurement of the bioactive component of the protein fraction content was performed using an RP-HPLC Agilent 1100 instrument and a Jupiter 5u C18 300A column according to the methodology of Łukasiewicz et al. [8]. 

The determination of MDA (malondialdehyde) was carried out using a Tecan NanoQuant Infinite M200 PRO analyzer (Tecan Austria GmbH, Grödig, Austria) according to the methodology of Kapusta et al. [16]. 

The cholesterol determination was achieved using an Agilent 7890A gas chromatograph (Agilent Technologies, Waldbronn, Germany) and a BP-5 column according to the methodology of Kapusta et al. [16]. 

DAP (degree of antioxidant protection) was calculated from the molar ratio between antioxidants and oxidants according to Pizzoferrato et al. [17]:

$$DAP=\frac{\sum_{i=1}^{n}AC_{i}\;(n^{{}^{\circ}\;}moles)}{OT\;(n^{{}^{\circ}}moles)}$$

Total antioxidant potential (TAS) according to RANDOX application.

Incubation of ABTS^®^ with peroxidase (methemoglobin) leads to the formation of radical cation ABTS + +. This substance is blue-green and can be detected at 600 nm. Antioxidants present in the sample reduce the formation of blue-green color in proportion to their concentration.

HX-Fe^III^ + H_2_O_2_ → X-[Fe^IV^ = 0] + H_2_O

ABTS^®^ + X-[Fe^IV^ = 0] → ABTS^®+^ + HX-Fe^III^

where HX-Fe^III^—metmyoglobin, X-[Fe^IV^ = 0], ABTS^®^—2,2-azino-di [3-ethylbenzothiazolinosulfonate] (RANDOX materials). U/L defines the concentration of TAS.

### 2.2. Statistical Analysis

The data obtained were subjected to analysis of variance using ANOVA. The distribution of bioactive components was checked using the Shapiro–Wilk test. All tests were performed using the IBM SPSS 23 (2023) package [18]. Data were presented as least-squares means with standard error of the mean.

The following statistical model was used:

Y*ijk* = µ + A*i* + e*ij*

where *Yijk*—value of the tested trait; µ—mean; A*i*—effect of the *i*-th breed (*i* = 1–4); *eij*—standard error.

## 3. Results

For the most part, the results in Table 4 show significant differences between the protein, crude fat, and collagen content of muscle. The highest proportion of protein was determined for the LM breed, and was 22.32% higher than that of the PHF breed, which had the lowest protein content. Protein is the most valuable nutrient, so the highest possible results should be expected in resource-consuming meat production. 

The fatty acid profile showed significantly better results for LM, CH, and HH compared with PHF in the C18:2 *cis*-9, *trans*-11 group, which is a potent antioxidant and may have anti-carcinogenic effects (Table 5). The results of the fatty acid profile were higher for HH (40.15%) and CH (64.86%). These are important differences that should be taken into consideration in consumer decisions for their health-promoting properties [19]. The greatest differences in the analyzed acids were observed in the C22:6 and C18:1 content in the CH group compared with PHF, with differences of 100% and 96.39%, respectively. Fatty acid content in the PHF breed was no higher compared to the rest. These results indicate that beef breeds have a very significant advantage over dairy breeds. 

β-Carotene and α-retinol, which function as vitamin A, showed high variability across breeds (Table 6). The lowest results were observed for PHF in each group. The smallest difference was found between HH and PHF for α-retinol, with a variation of 3.03%, and the highest was between LM and PHF for β-carotene, at 80%. Large differences were observed in the α-tocopherol group, which is a type of vitamin E with strong antioxidant properties. CH had a 93.17% higher result relative to PHF, while LM showed the smallest difference, but still high, at 72.67% relative to PHF (Table 6). In most of the analyzed cases, the differences were significant and reached dozens of percentage points, and in some cases exceeded 90%, which indicates the significantly higher amounts of vitamin content in the meat of beef breeds compared with that of the dairy PHF breed. 

The highest total antioxidant status (TAS) was found in CH and was 147.5% higher than PHF. This is a very large difference, and indicates that the meat of this breed has significantly greater antioxidant properties compared with that of the other breeds (Table 7). Also, compared with PHF, a large difference of 70% was observed in LM, while in HH it was only 6.25%. For the degree of antioxidant potential (DAP), the highest concentration was found in LM, while CH had a slightly lower score than LM. Both the TAS and DAP indexes indicate antioxidant properties, and it is desirable to have them at their highest possible concentrations. In the case of malondialdehyde (MDA), the lower the score, the better. The lowest MDA content was found in CH, with LM being only slightly worse, while in HH the result was significantly worse. For all three analyzed elements, PHF had the worst results, indicating that it had lower quality and lower nutritional value parameters (Table 7). 

The analysis of the bioactive content of the protein fraction was in line with previous results. The higher the index of each parameter, the higher the nutritional value of the meat. PHF had the lowest scores for each of the analyzed parameters (Table 8). For anserine, taurine, creatinine, and creatine content, the highest results were found for LM. For carnosine and coenzyme Q10, the highest values were found for CH. In the HH group, all analyzed elements were at higher levels than the PHF group, but they were not as high as they were in the LM and CH groups (Table 8).

## 4. Discussion

Long et al. [20] conducted research revealing that optimal slaughter ages and weights vary significantly depending on the rate of maturity, which is characterized by the accumulation of fat during the “finishing” period. The average fattening time has been standardized at 605 days. PHF gained 0.82 kg per day, LM 1.08 kg, CH 1.06, and HH 1.04 kg (Table 2). Sakowski et al. [21], Kayar and İnal [22], and Pogorzelska et al. [23] reported similar trends in fattening rates and fattening results for LM, CH, and HH. Southgate et al. [24] conducted a study comparing breeds slaughtered at the same carcass fat cover. They found that Canadian Holsteins required approximately an additional 65 and 45 days to reach slaughter in a 16-month and 24-month production system, respectively, compared with either British Friesian or Charolais × Friesian steers. The Netto daily gains (daily carcass gains, Table 2) of PHF were 0.53 kg, 0.68 kg for LM, 0.67 kg for CH, and 0.66 for HH; this stays in agreement with McGee et al. [25] and indicates the benefits of CH steers compared with HF steers in fattening. Those results show the lowest daily gains and daily carcass gains for PHF achieved on the same feeding. 

The growth ability of cattle is influenced by various factors, including breed, genetic predisposition, nutrition, microclimatic conditions, farm or breeding conditions, and the month of birth [26]. Daily weight gains can be visualized using a growth curve, which exhibits a sigmoid character [27]. Differences in growth ability exist not only among different breeds but also among individuals within the same breed, emphasizing the impact of body frame on slaughter age and carcass weight [28]. The length of the fattening period significantly affects growth parameters and carcass quality. Ustuner et al. [29] confirmed that both the initial weight at the start of fattening and the timing for the end of the fattening period are crucial for the final meat production performance. Carcass weights were 536 kg, 694 kg, 689 kg, and 669 kg for PHF, Limousine, Charolaise, and Herford breeds, respectively (Table 2). The differences in standardized live weight among these breeds can be attributed to various factors, including genetic characteristics and growth rates. Albertí et al. [30] reported that LM and CH breeds had higher carcass yields than Angus and HH. However, HH had the highest slaughter weight. Each breed has its own genetic potential for growth and carcass development, which influences the final carcass weight attained. Abramowicz et al. [31] highlighted that fat accumulation occurs after the relative growth of muscle decreases, while bone growth continues to decrease. This suggests that as animals mature, there is a shift in nutrient allocation towards fat deposition. The growth rate of fatty tissues can vary depending on their location and the stage of growth [32]. This indicates that different fat depots may exhibit different growth patterns and rates. In the study by Berg et al. [33], the carcass composition of seven different beef breeds was compared. The breeds exhibited variations in the muscle, fat, and bone composition of the carcasses. Notably, larger-framed breeds such as Chiannia and Blonde d’Aquataine resulted in carcasses with less fat compared with Danish Red and Hereford at a standard carcass weight. This suggests that breed-specific characteristics play a significant role in determining carcass composition.

Augustini et al. [34] demonstrated that the percentage of carcass meat and the proportion of beef cuts undergo changes during the growth of cattle. As animals mature, each tissue reaches its growth maximum at different stages, resulting in alterations in carcass tissue composition. Irshad et al. [35] highlighted that late-maturing cattle breeds exhibit slower physiological development compared with early-maturing breeds. This slower development is associated with a higher growth potential and slower fat accretion. Late-maturing breeds tend to show a preference for leaner carcasses, as they exhibit faster growth rates and more efficient conversion of high-energy feed into carcass weight. Van der Westhuizen [36] supported the notion that late maturity in cattle leads to increased growth of leaner carcasses. The delayed maturity allows for faster growth rates and improved conversion of feed into carcass weight [6]. This suggests that late-maturing breeds may have advantages in terms of producing leaner beef. The highest proportion of fat was determined for the HH breed, and was 2.04% higher than that of the PHF breed (Table 4). The Limousine breed had the highest average standardized live weight of 694 kg, followed closely by the Charolais breed with 689 kg. These breeds are known for their excellent muscling and growth potential, which may contribute to their higher carcass weights. The Hereford breed had a slightly lower average standardized live weight of 669 kg (Table 4). Collectively, these studies emphasize the dynamic nature of tissue development during cattle growth and the impact of maturity on carcass composition. Kempster et al. [37] highlight that when cattle of diverse breeds are compared at the same age and under similar management conditions, there will be variations in carcass weight. These differences are influenced by various factors, including the breed-specific growth curves and the range of target weights for each breed.

The results of the basic chemical composition, functional fatty acids, bioactive components of the protein fraction, fat-soluble vitamins, and oxidative and antioxidant potential indicate that PHF beef will have a significantly lower palatability and nutritional quality. Concerning fat content, LM and CH had significantly lower percentages (−26.78% and −23.39%, respectively) than the PHF breed. In the realm of cattle genetics and fat partitioning, Kempster et al. [37] conducted a seminal study demonstrating the significance of genetic variation in the distribution of fat between different depots. The findings of this study underscore the role of genetics in shaping fat deposition patterns in cattle. Additionally, Casasús et al. [38] reported that such genetic variation in fat partitioning persists even under similar nutritional conditions, further supporting the influence of genetic factors on fat distribution. Low fat content is characteristic for consumer needs in many developed countries, despite its lower palatability [39]. Only HH had a higher fat content, which was minimal at 2.04%. As the dominant breed in beef production worldwide, HH is characterized by high palatability because of its higher intramuscular fat content. The crude fat content positively influences the juiciness of the meat as intramuscular fat, but not as cover fat around the meat [40,41]. Additionally, Diler et al. [42] reported that muscle and fat type are essential sources of variations in the textural characteristics, sensory panel attributes, and fatty acid profile of meat from Holstein-Friesian bulls. The highest collagen content was found in PHF—in culinary beef production, this indicator should be as low as possible due to its negative effect on meat tenderness [43]. Increased collagen content also negatively affects meat quality [44,45], and significantly higher proportions of it were found in PHF. Compared with the beef breeds HH, CH, and LM, the dominant PHF breed significantly stands out in terms of its lower nutritional value and quality. Meat quality might be improved by crossbreeding with beef breeds [6,46]. This is a solution that will help make progress in shaping the quality of the beef produced in Poland and positively influence its health-promoting properties. 

The hormonal and metabolic distinctions between beef cattle and dairy breeds play a crucial role in determining their respective fat deposition tendencies. As elucidated by Kempster et al. [37], beef cattle exhibit a remarkable ability to convert nutrients predominantly into proteins. In contrast, dairy breeds, due to their unique hormonal and metabolic status, tend to deposit more intra-abdominal fat. The influence of breed and feed type shape the basic chemical composition and nutritional value of beef. The positive effect of the fatty acids’ improvement has been confirmed in a number of studies concerning the Limousine [47,48], Polish Holstein-Friesian [49,50], Charolaise [51,52], and Hereford breeds [53,54]. The authors of the studies demonstrated there is a variability in the fatty acid profile, indicating a benefit in nutritional value from the use of meat from breeds with better monounsaturated and polyunsaturated FA profiles. However, Sobczuk-Szul et al. [55] reported that intramuscular fat had a higher MUFA concentration (46.2%) than visceral fat (36.7%). Appropriate breed selection decreases the saturated FA content and improves the ratio of n-3 and n-6 fatty acids [21]. Barton et al. [56] indicated similar trends in the basic chemical composition of meat and the FA profile of LM and CH. Gregory et al. [57] pointed out that breed is an important factor in shaping slaughter performance. Comparative studies on the fattening value of PHF, LM, CH, and HH confirmed these reports [58,59,60]. Additionally, Sargentini et al. [61] and Humada et al. [62] reported that total SFA content did not significantly alter with increasing slaughter age. Breed has a significant effect on the fatty acid profile and how fat is deposited with age due to differences in intramuscular fat [47]. The proportion of unsaturated fatty acids in intramuscular fat increases with age [63,64]. By 18 months of age, meat palatability increases [65], accompanied by changes in soluble collagen structure [66]. The mechanism of these changes is not fully understood [67], but they are observable over the life of the cattle. Cattle slaughtered at the same weight, but of different breeds, can be characterized by different intramuscular fat content, which is deposited after the muscles’ growth phase, indicating different rates of breed development [68]. Research by Nürnberg et al. [69] on the dairy German Holstein and Belgian Blue breeds indicate relatively small modifications in the proportions of saturated fatty acids and n-3 between 18 and 25 months of cattle age, which may suggest a limited effect of age on the fatty acid profile after 18 months. 

Fat-soluble vitamins are an essential part of the human diet, which can be supplemented with beef that is rich in vitamins E and A. There are a number of properties of vitamins E and A, from protective effects on lipids [70] to improved health in people with Alzheimer’s [71] and cancer [72], but we can still observe deficiencies of those vitamins in human diets [73]. It seems reasonable to raise vitamin E levels in animals to benefit both animal welfare and the diets of meat consumers. The results in Table 6 show that breed has a significant effect on the formation of levels of β-carotene, α-retinol, and α-tocopherol, which may suggest the need for further research on the concentrations of these vitamins in beef meat. The results obtained in this study indicate that there are significantly higher concentrations of these vitamins in LM and CH relative to PHF, and, in the case of HH, that α-tocopherol was clearly higher than in PHF. This allows us to conclude that the LM and CH breeds are significantly more nutritious than PHF. In addition, vitamin ratios can be further improved by pasture grazing, which beef breeds are predisposed to utilize effectively [74]. The effect of age on vitamin E content is described in the study by Warren et al. [75]. In the case of Holstein-Friesian and Angus breeds, the differences in concentration of animals uniformly fed vitamin E concentrations on the example of Longissimus muscle at 14, 19, and 24 months were 1.3, 1.4, and 1.6 μg/g, respectively.

TAS results for LM and CH were characterized by high values; however, CH had the highest score. For DAP, the results for LM and CH were similar. Skaperda et al. [76] indicated increased antioxidant potential, which can be further increased by utilizing pasture grazing [77]. In contrast to TAS and DAP, where higher values benefit animal health, MDA content should be as low as possible [78]. In this case, LM, CH, and HH showed significantly lower values than PHF. Elevated MDA levels can negatively affect beef quality [79], so nutritional supplements containing selenium are used to improve this situation [79,80]. 

Anserine β-Alanyl-3-methyl-l-histidine is a dipeptide, a methyl-carnosine derivative. It consists of β-alanine and l-(*N*-methyl) histidine. With carnosine, it exhibits antioxidant activity, and high concentrations are found in skeletal muscle. The concentration is influenced by breed, sex, age, environment, and type of muscle [8]. Carnosine decreases MDA concentrations [81], reduces cellular aging processes [82], and can inhibit metmyoglobin formation [83]. The largest difference relative to PHF was found for anserine in LM and was 21%, while the smallest difference was between PHF and HH for carnosine and was 8.34% in favor of HH. The study by Watanabe et al. [84] shows that between 15 and 25 months of age there can be significant changes in the content of anserine and taurine in the *Longissimus dorsi muscle*. Such a relationship was not observed for carnosine. However, the authors indicate a significant effect of breed on each of the three and a variation in concentration in individual carcass elements [85]. 

Taurine is a 2-aminoethylsulfonic acid and is responsible for maintaining adequate leukocyte levels; its lack negatively affects the ability of neutrophils to oxygen burst and carry out phagocytosis. It also reduces the effects of oxidative stress and pro-inflammatory changes [86], and is involved in free radical neutralization, membrane stabilization, the formation of bile acid salts, and the maintenance of calcium homeostasis [87]. In this study, taurine was shown to have increased concentrations in all the beef breeds relative to PHF, reaching as high as 26.87% in LM. Taurine is also important for reducing nervous tension and improving mental performance by increasing glial cell metabolism [88]. It also has lipids, protective properties [89], and extends the shelf-life of beef [90]. 

Meat is a rich source of coenzyme Q10 [91]. It is an important component in the oxidative phosphorylation process. It converts energy from carbohydrates and fatty acids into ATP [92] and is part of the mitochondrial electron transport chain [93]. A lack of coenzyme Q10 results in the insufficient production of high-energy compounds [94]. A reduced form of coenzyme Q10 called ubiquinone is able to renew vitamin E [95]. The largest differences, when compared with PHF, were found in CH (35.82%), while in LM the variation was 24.60%, and that in HH was 11.23%. 

Creatinine is a creatine derivative formed during metabolic processes as an endogenous metabolite of the non-enzymatic breakdown of creatine phosphate. Creatinine is used to store and transfer energy in muscle cells and tendons. It is synthesized in the body; however, it must also be partially supplied via the human diet from food [96,97]. All the beef breeds in the study contained higher concentrations of creatinine relative to PHF: 36.17% for LM, 32.04% for CH, and 17.72% for HH. 

Creatine is synthesized from arginine, methionine, and glycine [98]. It is responsible for providing the energy in skeletal muscles, especially muscles characterized by a high energy transfer to ADP in muscle cells [99]. Creatine is converted to creatinine through the non-enzymatic processes of creatine phosphate breakdown. Its concentration in the samples showed less variation across all the breeds than did creatinine. It was higher than PHF by only 6.47% for LM, 5.36% for CH, and 3.55% for HH.

The quality of beef is significantly influenced by functional type and breed. Beef production in Poland is based on dairy or dual-purpose cattle herds. Intensive feeding and extended finishing time can favorably affect the yield and quality of beef produced. It is important to increase the intensity and method of feeding in the final fattening stage [100]. The high growth potential of the animal at a young age is inhibited when the animal reaches somatic maturity [101]. Late-maturing animals are effective for intensive fattening. The growth phase, where most fat is deposited, is postponed, so they can be fattened to high body weights without a decrease in carcass quality. Tissue development occurs through the expansion of the skeleton, followed by muscle and fat in the last stage. Age affects nutritional value, so slaughtering rules have been adopted to help maintain quality beef [102]. All animals were fed the same diet and slaughtered at around 20 months of age, which allowed the results to be used for comparison after standardization due to the effect of age on shaping beef quality and composition. From birth to physical maturity, the rate of muscle growth is higher than the rate of fat deposition and influence on fatty acid composition [103]. The effect of age on the development of the fatty acid profile is important and should be taken into account during the current work on beef quality. It also affects the vitamin content of the meat [58] and is an important element in the formation of beef quality [21]. This also affects the bioactive protein fraction [84]. Therefore, it is important to take into account the age of the animals in comparisons for different breeds and feeding systems. The age of the animal is an important factor shaping meat quality. With age, the fat content and size of fat cells change [69,104]. 

Each cattle breed possesses unique genetic characteristics that influence their growth potential and carcass development. As cattle mature, there is a shift in nutrient allocation, with a decrease in the relative growth of muscle and bone and an increase in fat accumulation. This phenomenon implies that as animals reach their mature stages, they tend to deposit more fat, contributing to the final carcass weight attained. The allocation of nutrients towards fat deposition during the finishing period is essential for meat production and quality. The balance between muscle and fat development can impact the meat’s marbling, tenderness, and flavor. Understanding breed-specific growth patterns and nutrient partitioning is crucial for the effective management and optimization of cattle production.

## 5. Conclusions

Overall, these results highlight the impact of maturity and breed on carcass composition and quality. Late-maturing breeds, such as LM and CH, tend to exhibit leaner carcasses with superior fatty acid profiles and antioxidant properties. The fatty acid profile showed significantly better results for LM and CH compared with HH and PHF in the C18:2 *cis*-9, *trans*-11 group, which is a potent antioxidant and may have anti-carcinogenic effects. Large differences were observed in the α-tocopherol group, which is a type of vitamin E with strong antioxidant properties. CH had a 93.17% higher result relative to PHF, while LM showed the smallest difference, but still high, at 72.67% relative to PHF. The highest TAS was found in CH and was 147.5% higher than PHF. Also, compared with PHF, a large difference of 70% was observed in LM, while in HH it was only 6.25%. For DAP, the highest concentration was found in LM, while CH had a slightly lower score than LM. Both the TAS and DAP indexes indicate antioxidant properties, and it is desirable to have them at their highest possible concentrations. The lowest MDA content was found in CH, with LM being only slightly worse, while in HH the result was significantly worse. PHF had the lowest scores for each of the analyzed parameters of protein fraction. For anserine, taurine, creatinine, and creatine content, the highest results were found for LM. For carnosine and coenzyme Q10, the highest values were found for CH. In the HH group, all analyzed elements were at higher levels than the PHF group, but they were not as high as they were in the LM and CH groups. 

Collectively, these studies emphasize the dynamic nature of tissue development during cattle growth and the impact of maturity on carcass composition. Late-maturing breeds exhibit a different growth pattern, with slower fat deposition and a preference for leaner carcasses. Carcass composition is a result of the interplay between genetic factors, growth patterns, and management practices. Different cattle breeds exhibit distinct growth trajectories and rates of fat deposition, which ultimately affect the composition of their carcasses. Additionally, the target weights set for each breed based on market preferences and production goals also play a role in determining the composition of the final carcass. This knowledge has implications for breed selection, production strategies, and meeting specific market demands for beef with desired composition and quality.

## Figures and Tables

**Table 1:** Population changes of PHF, LM, CH, and HH [4].

	No. of Heads in 2012	No. of Heads in 2021	Population Change
**PHF**	597,715	704,506	106,791 (+17.86%)
**Limousine**	11,879	13,948	+2069 (+17.4%)
**Charolaise**	2265	1571	−694 (−30.6%)
**Hereford**	743	1409	+666 (+89.6%)

**Table 2:** Bull characteristics on the day of slaughter (standardized at 605 days).

	Number (*n*)	Standardized Live Weight (kg)	Carcass Weight (kg)	Standardized Daily Gains (kg)	Daily Carcass Gains (kg)
**PHF**	16	536	317	0.82	0.53
**Limousine**	18	694	413	1.08	0.68
**Charolaise**	17	689	416	1.06	0.67
**Hereford**	16	669	390	1.04	0.66
*p-value*		*0.001*	*0.001*	*0.001*	*0.001*
SEM		4.251	8.213	0.054	0.012

**Table 3:** Characteristics of the research material.

Composition	Value
Maize silage (%)	68
Barley (%)	29
Supplements (%)	3
**Nutritional value**	
Dry matter (%)	54
Protein (g/kg)	128
NEm (Mcal/kg)	1.77
Neg (Mcal/kg)	1.15
NDF (g/kg)	343
ADF (g/kg)	194
Crude fat (g/kg)	19

**Table 4:** The effect of breed on the formation of the basic chemical composition in *Semimembranosus muscles*. In parentheses are % of variation in relation to PHF. Means (in column) marked with the same letters differ significantly at: lowercase letters, *p* ≤ 0.05; uppercase letters, *p* ≤ 0.01.

	Protein [g/100 g]	Crude Fat [g/100 g]	Collagen [mg/100 g]
**PHF**	19.40 ^A,B,C^	2.95 ^A,B^	592.24 ^A,B,C^
**Limousine**	23.73 ^A,d^ (+22.32%)	2.16 ^A,C^ (−26.78%)	549.84 ^A,d^ (−7.16%)
**Charolaise**	22.05 ^B^ (+13.66%)	2.26 ^B,D^ (−23.39%)	542.52 ^B,e,^ (−8.40%)
**Hereford**	21.21 ^C,d^ (+9.33%)	3.01 ^C,D^ (+2.04%)	552.62 ^C,d,e^ (−6.69%)
SEM	1.236	0.441	7.625

**Table 5:** The effect of breed on the formation of functional fatty acid levels in *Semimembranosus muscles*. In parentheses are % of variation in relation to PHF. Means (in column) marked with the same letters differ significantly at: lowercase letters, *p* ≤ 0.05; uppercase letters, *p* ≤ 0.01.

[g/100 g]	C18:1 *trans*-11	C18:2 n-6	C18:2 *cis*-9, *trans*-11	C18:3 n-3	C20:5 n-3	C22:6 n-3
**PHF**	0.83 ^A,B,C^	8.24 ^A,B,C^	2.59 ^A,B,C^	0.49 ^A,B,C^	0.42 ^A,B,C^	0.07 ^A,B,C^
**Limousine**	1.39 ^A,D,E^ (+67.45%)	12.19 ^A,d,E^ (+47.94%)	4.03 ^A,d,E^ (+55.60%)	0.74 ^A,D^ (+51.2%)	0.71 ^A,D^ (+69.05%)	0.11 ^A,d^ (+57.14%)
**Charolaise**	1.63 ^B,D,F^ (+96.39%)	13.45 ^B,d,F^ (+63.23%)	4.27 ^B,d,F^ (+64.86%)	0.71 ^B,E^ (+44.90%)	0.74 ^B,E^ (+76.19%)	0.14 ^B,d,E^ (+100%)
**Hereford**	1.26 ^C,E,F^ (+51.80%)	10.38 ^C,E,F^ (+25.97%)	3.63 ^C,E,F^ (+40.15%)	0.66 ^C,D,E^ (+34.70%)	0.65 ^C,D,E^ (+54.76%)	0.09 ^C,D,E^ (+28.57%)
SEM	0.233	0.478	0.119	0.017	0.041	0.011

**Table 6:** The effect of breed on the formation of fat-soluble vitamin levels in *Semimembranosus muscles*. In parentheses are % of variation in relation to PHF. Means (in column) marked with the same letters differ significantly at: lowercase letters, *p* ≤ 0.05; uppercase letters, *p* ≤ 0.01.

[μg/g]	β-Carotene	α-Retinol	α-Tocopherol
**PHF**	0.20 ^A,B,C^	0.66 ^A,B,C^	1.61 ^A,B,C^
**Limousine**	0.36 ^A,D^ (+80%)	0.81 ^A,D^ (+22.73%)	2.78 ^A,D,E^ (+72.67%)
**Charolaise**	0.33 ^B,E^ (+65%)	0.79 ^B,E^ (+19.70%)	3.11 ^B,D,f^ (+93.17%)
**Hereford**	0.21 ^C,D,E^ (+5%)	0.68 ^C,D,E^ (+3.03%)	3.08 ^C,E,f^ (+91.30%)
SEM	0.012	0.078	0.113

**Table 7:** The effect of breed on the formation of TAS, DAP, and MDA levels in *Semimembranosus muscles*. In parentheses are % of variation in relation to PHF. Means (in column) marked with the same letters differ significantly at: lowercase letters, *p* ≤ 0.05; uppercase letters, *p* ≤ 0.01.

	TAS [mmol/L]	DAP	MDA [mM/g]
**PHF**	0.80 ^A,B,C^	5.12 × 10^−3^ ^A,B,C^	3.30 ^A,B,C^
**Limousine**	1.36 ^A,D,E^ (+70%)	8.10 × 10^−3^ ^A,d,E^ (+58.20%)	1.24 ^A,D^ (−62.42%)
**Charolaise**	1.98 ^B,D,F^ (+147.5%)	7.80 × 10^−3 B,d,F^ (+52.34%)	1.20 ^B,E^ (−63.64%)
**Hereford**	0.85 ^C,E,F^ (+6.25%)	6.25 × 10^−3^ ^C,E,F^ (+22.07%)	2.45 ^C,D,E^ (−25.76%)
SEM	0.011	0.013	0.013

**Table 8:** The influence of breed on bioactive protein fraction levels in *Semimembranosus muscles*. In parentheses are % of variation in relation to PHF. Means (in column) marked with the same letters differ significantly at: lowercase letters, *p* ≤ 0.05; uppercase letters, *p* ≤ 0.01.

[mg/100 g]	Anserine	Carnosine	Taurine	Coenzyme Q10	Creatinine	Creatine
**PHF**	61.22 ^A,B,C^	387.30 ^A,B,C^	34.28 ^A,B,C^	1.87 ^A,B,C^	4.12 ^A,B,C^	396.96 ^A,B,C^
**Limousine**	74.08 ^A,d,E^ (+21.00%)	431.53 ^A,D,E^ (+11.42%)	43.49 ^A,d,E^ (+26.87%)	2.33 ^A,d,e^ (+24.60%)	5.61 ^A,d,E^ (+36.17%)	422.66 ^A,d,E^ (+6.47%)
**Charolaise**	72.52 ^B,d,F^ (+18.46%)	445.36 ^B,D,F^ (+14.99%)	42.14 ^B,d,F^ (+22.92%)	2.54 ^B,d,F^ (+35.82%)	5.44 ^B,d,F^ (+32.04%)	418.22 ^B,d,F^ (+5.36%)
**Hereford**	69.29 ^C,E,F^ (+13.18%)	419.59 ^C,E,F^ (+8.34%)	37.31 ^C,E,F^ (+8.84%)	2.08 ^C,e,F^ (+11.23%)	4.85 ^C,E,F^ (+17.72%)	411.05 ^C,E,F^ (+3.55%)
SEM	0.752	1.114	0.442	0.022	0.073	0.812

## Data Availability

All data generated or analyzed during the study are included in this published article. The datasets used and/or analyzed in the current study are available from the corresponding author on reasonable request.
